# The impact of pharmacist interventions, follow-up frequency and default on glycemic control in Diabetes Medication Therapy Adherence Clinic program: a multicenter study in Malaysia

**DOI:** 10.1186/s40545-023-00583-8

**Published:** 2023-07-05

**Authors:** Phei Ching Lim, Hooi Hoon Tan, Nurul Ain Mohd Noor, Chee Tao Chang, Te Ying Wong, Ee Linn Tan, Chiou Ting Ong, Kalyhani Nagapa, Lee Shyong Tai, Wei Ping Chan, Yong Boey Sin, Yin Shan Tan, Shanty Velaiutham, Rohaizan Mohd Hanafiah

**Affiliations:** 1 Pharmacy Department, Hospital Pulau Pinang, Ministry of Health Malaysia, George Town, Malaysia; 2 Pharmacy Department, Northeast District Health Office, Penang, Ministry of Health Malaysia, George Town, Malaysia; 3 Pharmacy Department, Hospital Balik Pulau, Ministry of Health Malaysia, Balik Pulau, Malaysia; 4 Clinical Research Centre, Hospital Raja Permaisuri Bainun, Ministry of Health Malaysia, Ipoh, Malaysia; 5 School of Pharmacy, Monash University Malaysia, Subang Jaya, Malaysia; 6 Pharmacy Department, Hospital Bukit Mertajam, Ministry of Health Malaysia, Bukit Mertajam, Malaysia; 7 Pharmacy Department, Hospital Sungai Bakap, Ministry of Health Malaysia, Sungai Jawi, Malaysia; 8 Pharmacy Department, Hospital Seberang Jaya, Ministry of Health Malaysia, Perai, Malaysia; 9 Pharmacy Department, Southwest District Health Office, Penang, Ministry of Health Malaysia, Balik Pulau, Malaysia; 10 Pharmacy Department, North District Health Office, Seberang Perai, Ministry of Health Malaysia, Kepala Batas, Malaysia; 11 Pharmacy Department, Center District Health Office, Seberang Perai, Ministry of Health Malaysia, Bukit Mertajam, Malaysia; 12 Pharmacy Department, South District Health Office, Seberang Perai, Ministry of Health Malaysia, Nibong Tebal, Malaysia; 13 Medical Department, Hospital Pulau Pinang, Ministry of Health Malaysia, George Town, Malaysia; 14 Penang Pharmaceutical Services Division, Ministry of Health Malaysia, George Town, Malaysia; 15 School of Pharmaceutical Sciences, University Science Malaysia, Gelugor, Malaysia

**Keywords:** Pharmacists, Diabetes, Interventions, Visits, Default, Glycemic control, HbA1c, Malaysia

## Abstract

**Background:**

Pharmacist’s involvement in optimizing medication adherence among diabetic patients has been implemented for over a decade. Diabetes Medication Therapy Adherence Clinic (DMTAC) was set up to educate diabetic patients, monitor treatment outcomes, and manage drug-related problems. While evidence shows that pharmacist-led DMTAC was effective in reducing HbA1c, there was limited data regarding the impact of different intervention types and default to follow-up on glycemic control.

**Aim:**

To assess the impact DMTAC on glycemic control and the difference in glycemic control between hospital and health clinic settings as well as defaulter and non-defaulter. In addition, the impact of pharmacist’s interventions, DMTAC follow-up frequencies, and duration of diabetes on glycemic control were also determined.

**Methods:**

A retrospective study was conducted among diabetes patients under DMTAC care between January 2019 and June 2020 in five hospitals and 23 primary health clinics. Patients’ demographics data, treatment regimens, frequencies of DMTAC visits, defaulter (absent from DMTAC visits) and types of pharmacists’ intervention were retrieved from patients’ medical records and electronic database. HbA1c was collected at baseline, 4–6 months (post-1), and 8–12 months (post-2).

**Results:**

We included 956 patients, of which 60% were females with a median age of 58.0 (IQR: 5.0) years. Overall, the HbA1c reduced significantly from baseline (median: 10.2, IQR: 3.0) to post-1 (median: 8.8, IQR: 2.7) and post-2 (median: 8.3, IQR: 2.6%) (*p* < 0.001). There were 4317 pharmacists’ interventions performed, with the majority being dosage adjustment (*n* = 2407, 55.8%), followed by lab investigations (849, 19.7%), drugs addition (653, 15.1%), drugs discontinuation (408, 9.5%). Patients treated in hospitals received significantly more interventions than those treated in primary health clinics (*p* < 0.001). We observed significantly less reduction in HbA1c in DMTAC follow-up defaulters than non-defaulters after 1 year (− 1.02% vs. − 2.14%, *p* = 0.001). Frequencies of DMTAC visits (*b*: 0.19, CI: 0.079–0.302, *p* = 0.001), number of dosage adjustments (*b*: 0.83, CI: 0.015–0.151, *p* = 0.018) and number of additional drugs recommended (*b*: 0.37, CI: 0.049–0.691, *p* = 0.024) had positive impact on glycemic control whereas duration of diabetes (b: − 0.0302, CI: − 0.0507, − 0.007, *p* = 0.011) had negative impact.

**Conclusion:**

Glycemic control improved significantly and sustained up to one year among patients in pharmacists-led DMTAC. However, DMTAC defaulters experienced poorer glycemic control. Considering more frequent visits and targeted interventions by pharmacists at DMTAC resulted in improved HbA1c control, these strategies should be taken into account for future program planning.

## Background

Diabetes is a global public health concern and one of the top ten causes of mortality worldwide [1]. According to International Diabetes Federation (IDF), there is a continual increase in diabetes prevalence globally, with an estimated 537 million adults living with diabetes in 2021; more than 75% were from low and middle-income countries [2]. Diabetes was associated with an increased risk of mortality due to complications from infections, cardiovascular disease, stroke, and chronic kidney disease and accounted for more than 6.7 million deaths in 2021 [2]. In Malaysia, the prevalence of diabetes in the adult population was 18.3% in 2019, which amounted to 3.9 million adults living with diabetes [3].

Most diabetes patients receive treatment in primary and tertiary care institutions in Malaysia. A multidisciplinary team approach has long been proven effective in improving diabetes health outcomes [4]. Over the years, pharmacists’ roles have expanded beyond the traditional screening, filling, and dispensing roles. Specifically, pharmacists play an integral role in diabetes management, as they are one of the most accessible healthcare professionals in the primary care setting.

In a study conducted in a community setting in the United States, pharmacist education and interventions resulted in a 1.3% decrease in glycosylated hemoglobin (HbA1c) [5]. Meanwhile, in a similar setting in Australia, a more significant proportion of diabetic patients achieve HbA1c < 7% following a 9-month intervention period by pharmacists compared to the control group. In addition to HbA1c reduction, patients' well-being and adherence improved [6]. A meta-analysis further consolidates the evidence that pharmacists’ interventions could improve HbA1c, blood pressure, and body mass index (BMI) in diabetic patients [7].

Since 2006, a diabetic outpatient program called Diabetes Medication Therapy Adherence Clinic (DMTAC) has been run by pharmacists in hospitals and health clinics in Malaysia. DMTAC pharmacists must undergo a structured training program within the Ministry of Health Malaysia before providing services. Patients enrolled in the service are required to visit the pharmacists at an average interval of one month for medication and disease education, treatment outcome monitoring, identification, and management of drug-related problems [8].

Previous local studies had reported positive outcomes in pharmacists-led DMTAC service. In the Penang state of Malaysia, a study by Lim et al. reported a mean HbA1c reduction of 1.73% and fasting plasma glucose (FPG) improvement of 2.65 mmol/L following the completion of 8 DMTAC visits in a tertiary hospital [9]. Another study in a university hospital reported HbA1c reduction from 9.66% to 8.47% in patients who received pharmacist interventions [10]. Significant HbA1c reductions were also observed among patients in district hospitals after one-year follow-up in a pharmacist-led diabetes program [11]. Moreover, the multi-center retrospective study that involved 14 health clinics in Malaysia showed 1% reduction in HbA1c among 56 type 2 diabetes mellitus patients [12].

Although several studies observed HbA1c reduction in patients with diabetes undergoing DMTAC program, most were conducted either in hospital or primary health clinic settings. Hence, there was a lack of evidence on the glycemic outcome among DMTAC patients in hospital as compared to primary health clinic settings. Moreover, data was limited regarding the types of intervention performed by pharmacists, frequency of DMTAC visits, and default rate. In this study, we aimed to evaluate the impact of DMTAC on glycemic control and the difference in glycemic control between hospitals and primary health clinics as well as between defaulter and non-defaulters. We also aimed to determine the impact of pharmacist’ interventions, the frequency of DMTAC visits and duration of diabetes on glycemic control among diabetic patients.

## Methods

This multicenter, retrospective study was conducted in all public healthcare facilities within the state of Penang, Malaysia, including five hospitals and 23 primary health clinics. This study was registered in National Medical Research Registry (NMRR-20-414-53115) and obtained ethical approval from the Medical Research Ethnics Committee, Ministry of Health Malaysia. All patients under the care of the Diabetes Medication Therapy Adherence Clinic (DMTAC) between January 2019 to June 2020 in the selected study sites were screened for eligibility. We included patients with diabetes mellitus aged 18 years and above recruited into DMTAC and excluded pregnant women, patients with end stage renal failure (eGFR < 15 mL/min/m^2^) and those with missing data.

DMTAC was operated based on Diabetes Medication Therapy Adherence Protocol by Pharmaceutical Services Division, Ministry of Health Malaysia [8]. The insulin titration or dose adjustment was performed based on Practical Guide to Insulin Therapy in Type 2 Diabetes Mellitus and Clinical Practice Guideline Management of Type 2 Diabetes Mellitus [13, 14]. Several interventions were undertaken within the DMTAC, encompassing medication initiation or discontinuations, dosage adjustments, and recommendations for laboratory investigations. Notably, the pharmacists were granted authorization by the physicians to carry out insulin dosage adjustments during the DMTAC sessions that occurred between the physicians' follow-up visits, while other interventions took place during the regular physician appointments.

DMTAC pharmacists routinely documented patients’ medical, social, and family histories, drug-related issues, interventions, laboratory data, treatment regimens, and adherence using the Malaysian Medication Adherence Tool (MyMAAT) score within the Pharmacy Information System (PhIS), an electronic database. The retrospective study data were retrieved from the patient medical records and the (PhIS). Patients' demographic information, treatment regimens, types of healthcare facilities, frequencies of DMTAC visits, default to follow-up rates, and details of pharmacists’ interventions, including recommendations to add or discontinue medications, dosage adjustments, and suggestions for laboratory investigations, were collected and recorded in a standardized electronic data collection form.

Default to follow-up was defined as absence in all DMTAC follow-ups between physician’s follow-ups, where patients only met DMTAC pharmacists during the doctor’s follow-up. The primary outcome was HbA1c level and were collected at baseline, 4–6 months (post-1), and 8–12 months (post-2). The secondary outcomes were the difference in HbA1c between diabetic patients in hospital and primary health clinic settings, difference in HbA1c between defaulter and non-defaulter and the impact of pharmacist’ interventions, the frequency of DMTAC visits, and duration of diabetes on HbA1c.

Convenience sampling with proportionate representation was utilized in this retrospective study. Patients were selected based on their availability in medical records, and a higher number of subjects were included from sites with larger patient populations. The sample size was determined using PS software version 3.1.6, employing a one-sided paired Z-test. A significance level (alpha) of 0.05 and a power of 0.9 were assumed. The data were expected to follow a normal distribution with a standard deviation of 1.7 [12]. Based on an estimated difference in mean HbA1c of 0.5%, a sample size of 123 patients was calculated to be required to reject the null hypothesis. Considering an anticipated dropout rate of 20%, a total sample size of 148 patients was derived. The chosen Type I error probability for this test of the null hypothesis was 0.05.

The data were analyzed using IBM Statistical Package for Social Sciences (SPSS) software Version 16.0. Data in categorical values were presented in frequency and analyzed using Chi-square. The HbA1c data was not normally distributed for all patients and comparison between hospitals and primary health clinics. Hence, Wilcoxon signed rank test and Mann–Whitney *U* test were used for within and between groups comparison respectively. Meanwhile, paired t-tests and independent t-tests were used for within and between groups comparison for the HbA1c data for defaulter and non-defaulters as the data was normally distributed. The relationship between difference in HbA1c and number of DMTAC visits, pharmacist’ interventions and duration of diabetes were analyzed using linear regression analysis. Results were significant if *p*-values were less than 0.05.

## Results

956 subjects were analyzed, including 395 from hospitals and 561 from health clinics. The median age of the patients was 58 years old (IQR: 15), with 574 females (60.0%) and 407 Malays (42.6%). Compared to health clinics, hospital patients missed more DMTAC appointments (21.8% vs. 12.3%, *p* < 0.001). However, significantly more pharmacists’ interventions were performed in hospitals compared to health clinics (median 5.0 vs. 3.0, *p* < 0.001).

819 patients had dyslipidemia (85.7%), followed by 748 with hypertension (78.2%), 184 with cardiovascular disease (19.2%), 253 with neuropathy (26.5%), 227 with nephropathy (23.7%) and 202 with retinopathy (21.1%).

781 patients (81.7%) recruited in DMTAC had insulin treatment. The proportion of patients who received at least one type of insulin at health clinics (87.5%) was higher than those in hospitals (72.7%). Meanwhile, patients who visited hospitals received more interventions than those in health clinics [hospital median: 5 (IQR: 4), health clinics median: 3 (IQR: 5), *p* < 0.001] (Table 1).

**Table 1:** Demographic characteristics of subjects

Demographic characteristic	Number of patients, *n* (%)			*p*-value
	Total patients (*n* = 956)	Hospital (*n* = 395)	Primary health clinic (*n* = 561)	
Gender				
Male	382 (40.0)	173 (43.8)	209 (37.3)	0.042^a^
Female	574 (60.0)	222 (56.2)	352 (62.7)	
Age (year)	58 (15.0)	57 (17.0)	59 (13.0)	0.003^b^
Race				
Malay	407 (42.6)	157 (39.7)	250 (44.6)	0.258^a^
Chinese	322 (33.7)	132 (33.4)	190 (33.9)	
Indian	220 (23.0)	102 (26.1)	117 (20.9)	
Other	7 (0.7)	3 (0.8)	4 (0.7)	
Duration of DM (year)	10 (10.0)	10 (10.0)	10 (8.0)	0.625^b^
Body Mass Index, BMI (kg/m^2^)	26.9 (6.6)	27.1 (6.8)	26.8 (6.4)	0.816^b^
Default follow-up				
Yes	155 (16.2)	86 (21.8)	69 (12.3)	< 0.001^a^
No	801 (83.8)	309 (78.2)	492 (87.7)	
Total interventions (median, IQR)	4.0 (5.0)	5.0 (4.0)	3.0 (5.0)	< 0.001^b^
Total visits to post 1 (median, IQR)	4.0 (2.0)	4.0 (1.0)	4.0 (3.0)	0.044^b^
Total visits post 1 to post 2 (median, IQR)	5.0 (4.0)	4.0 (4.0)	7.0 (5.0)	< 0.001^b^
Comorbid				
Dyslipidemia	819 (85.7)	326 (82.5)	493 (87.9)	0.020
Hypertension	748 (78.2)	299 (75.7)	449 (80.0)	0.109
* Macrovascular*				
Cardiovascular disease	184 (19.2)	102 (25.8)	82 (14.6)	< 0.001
Peripheral vascular disease	36 (3.8)	20 (5.1)	16 (2.9)	0.077
Cerebrovascular disease	32 (3.3)	14 (3.5)	18 (3.2)	0.776
* Microvascular*				
Neuropathy	253 (26.5)	140 (35.4)	113 (20.2)	< 0.001
Nephropathy	227 (23.7)	94 (23.8)	133 (23.7)	0.970
Retinopathy	202 (21.1)	74 (18.7)	128 (22.8)	0.128
Type of regimen				
Single OGLD*	26 (2.7)	22 (5.6)	4 (0.7)	< 0.001
Two OGLD	137 (14.3)	73 (18.5)	64 (11.4)	
Triple OGLD	11 (1.2)	9 (2.3)	2 (0.4)	
OGLD + basal insulin	228 (23.8)	76 (19.2)	152 (27.1)	
OGLD + premixed insulin	336 (35.1)	131 (33.2)	205 (36.5)	
OGLD + basal bolus	135 (14.1)	46 (11.6)	89 (15.9)	
Insulin only	83 (8.7)	38 (9.6)	45 (8.0)	

^a^Data was in frequency and analysis using Chi−square ^b^Data was in median (IQR) and analysis using Mann–Whitney *U* test

*OGLD oral glucose-lowering drug

**Fig. 1:**
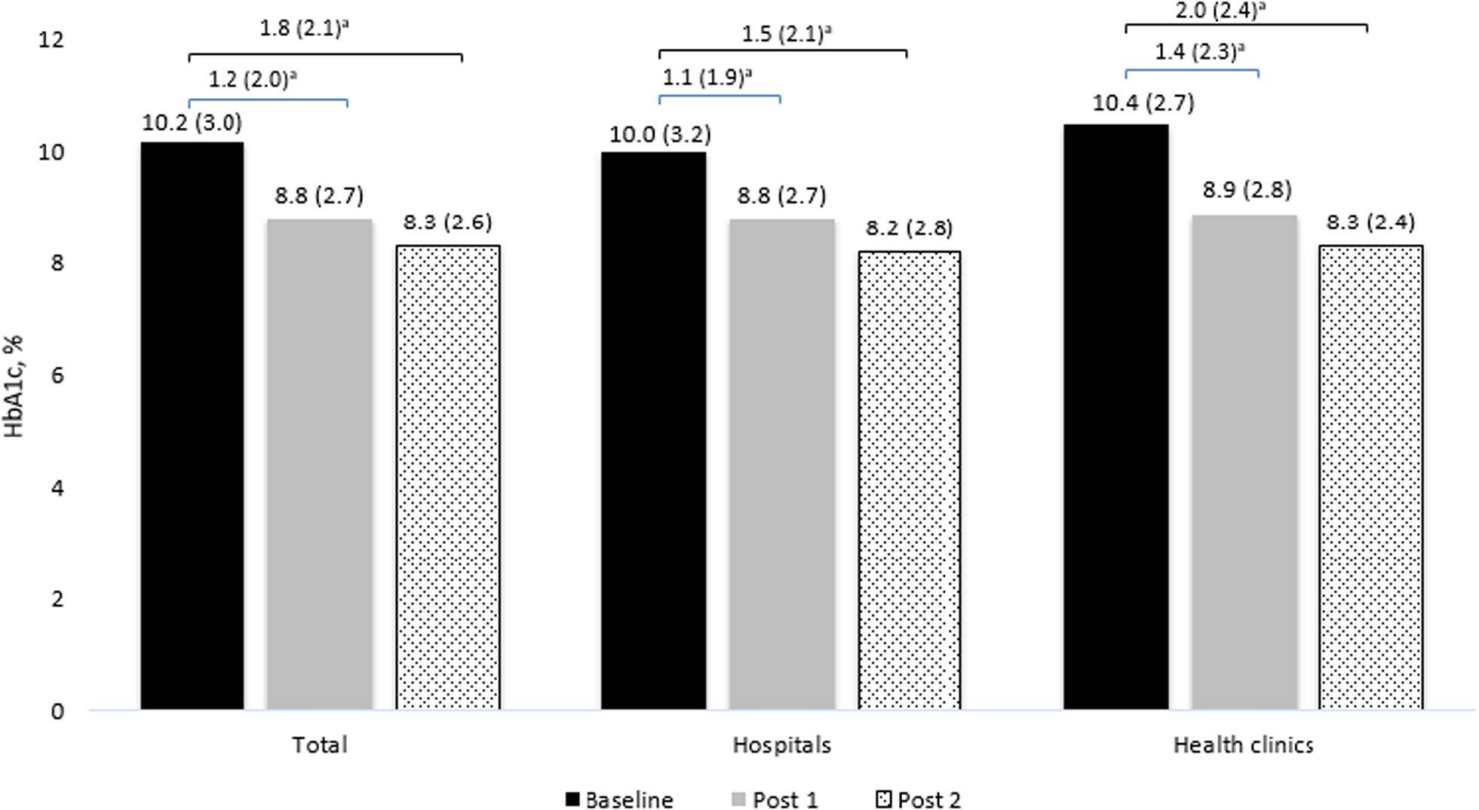
HBAIC differences at baseline, post-1, and post-2 between hospitals and health clinics. Data that was not normally distributed was presented in Median (IQR). ^a^Analysis using Wilcoxon signed rank test and significantly different, *p* < 0.001

HbA1c reduced significantly among patients recruited in DMTAC in hospitals and health clinics from baseline to post 1 and post 2 (Fig. 1). However, there was no significant difference in HbA1c improvement between hospitals and health clinics from baseline to post 1 (− 1.1% vs. − 1.4%, *p* = 0.062) and baseline to post 2 (− 1.5% vs. 2.0%, *p* = 0.058), respectively.

**Fig. 2:**
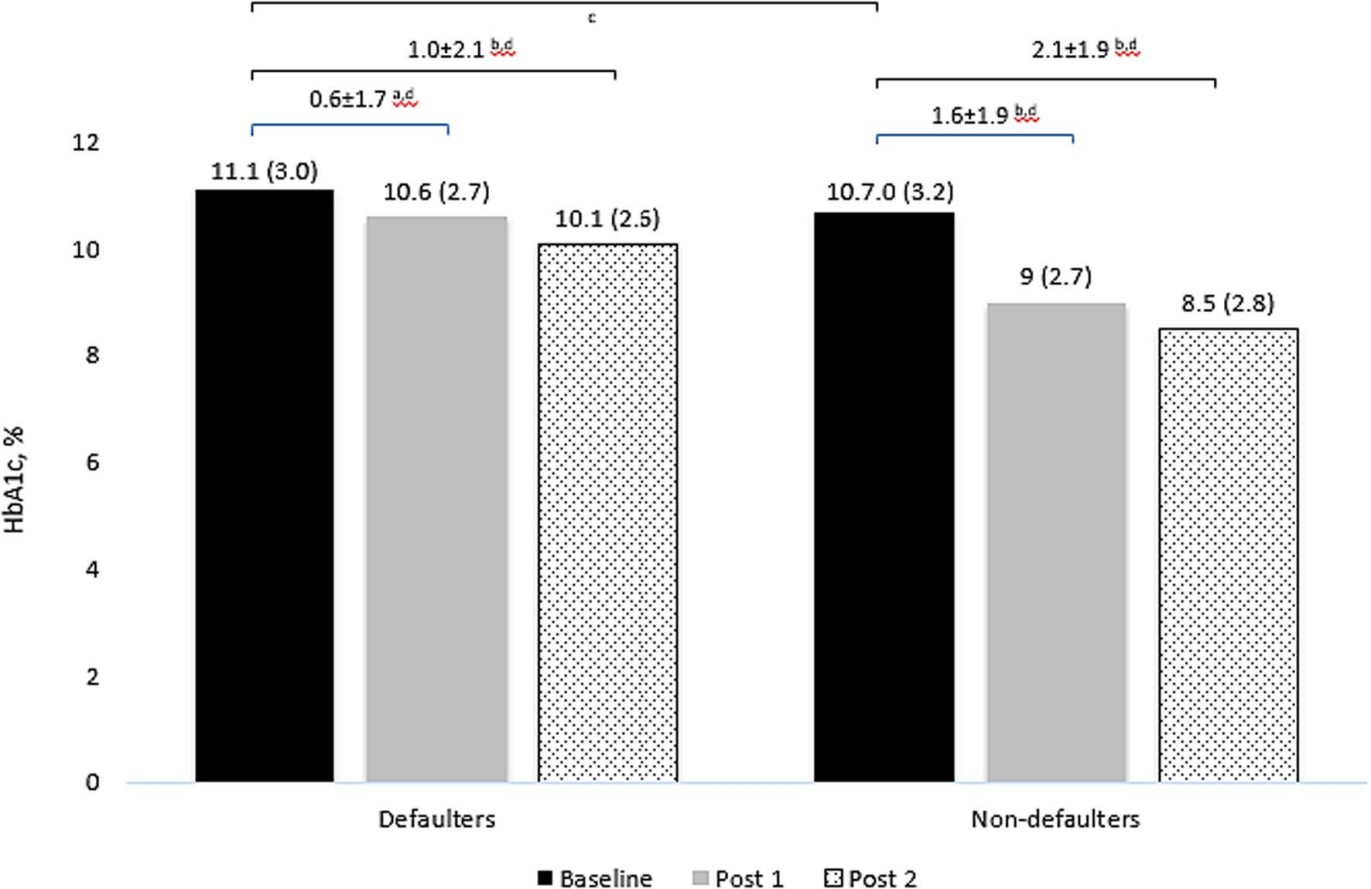
HBAIC differences at baseline, post-1, and post-2 between defaulters and non-defaulters. Data that was normally distributed was presented in Mean ± SD. ^a^Analysis using paired *t*-test and not significantly different, *p* > 0.05. ^b^Analysis using paired *t*-test and significantly different, *p* < 0.05. ^c^Analysis using independent *t*-test and not significantly different, *p* = 0.226. ^d^Analysis using independent *t*-test and significantly different, *p* = 0.001

At baseline, there was no difference in HbA1c between defaulters and non-defaulters (11.1 vs. 10.7, *p* = 0.226) (Fig. 2). In post 1, HbA1c improved significantly from baseline (− 1.63%, *p* < 0.001) among non-defaulters, and there was no significant difference among defaulters (− 0.57%, *p* = 0.05). In post 2, even though HbA1c reduced significantly among defaulters from baseline (− 1.02%, *p* = 0.006), non-defaulters had a significantly higher magnitude of improvement (− 2.14, *p* = 0.001) (Fig. 2). Other factors like gender, race, type of regimen, and type of facilities were not significantly associated with HbA1c reduction.

Out of the four types of interventions performed by DMTAC pharmacists, dosage adjustment was the most commonly suggested (55.8%), followed by lab investigations recommendations (19.7%), adding medications (15.1%) and medications discontinuation (9.5%) (Table 2).

**Table 2:** Types of Interventions Performed by Pharmacists in DMTAC

Intervention	Number of interventions, n	Percentage (%)
Adding medications (total)	653	15.0
OGLD	334	7.7
Statin	70	1.6
Anti-hypertensive drugs	78	1.8
Other medications	171	3.9
Discontinue medications	408	9.5
Dosage adjustment	2407	55.8
Recommend lab investigations	849	19.7
Total	4317	100

In the multiple linear regression model, frequencies of DMTAC visits from baseline to post 1 (*b* = 0.19, CI: 0.079–0.302, *p* = 0.001) and duration of DM (*b* = − 0.0302, CI: − 0.0507 to − 0.007, *p* = 0.011) were predicting factors of HbA1C reduction from baseline to post 1. The final model equation was: HbA1C reduction from baseline to post 1 = 1.15 + (0.19 × number of MTAC visit) − (0.032 × duration of diabetes), R^2^ = 0.042.

The frequencies of DMTAC visits did not predict HBAIC reduction from baseline to post 2 (*p* = 0.056). However, the number of dosage adjustments and the addition of anti-diabetic agent were associated with HbA1c differences from baseline to post 2 (Table 3). The final model equation was: HBAIC reduction from baseline to post 2 = 1.618 + (0.83 × number of dosage adjustment) + (0.37 × number of addition of antidiabetic agent), R^2^ = 0.040.

**Table 3:** Predicting factors of HbA1C differences in multiple linear regression model

Variables	Multiple linear regression	
	b (95% CI)	*p*-value
HbA1c reduction from baseline to post 1		
Frequencies of DMTAC visits from baseline to post 1	0.19 (0.079,0.302)	0.001
Duration of DM (year)	− 0.0302 (− 0.0507, − 0.007)	0.011
HbA1c reduction from baseline to post 2		
Number of dosage adjustments	0.83 (0.015,0.151)	0.018
Number of additional anti-diabetic agent	0.37 (0.049,0.691)	0.024

## Discussion

This study revealed a significant HbA1c reduction among diabetic patients after DMTAC visits. This was in line with two small-scale Malaysian studies conducted in a health clinic and a tertiary hospital, which demonstrated a smaller degree but significant HbA1c reduction [15, 16]. Within expectations, we observed that patients who did not default from follow-up achieved a significantly lower level of HbA1c in both time points than defaulters. Several other factors, such as the frequencies of DMTAC visits, duration of diabetes, number of dosage adjustments, and antidiabetic agents were identified as independent predictors of HbA1c levels.

Overall, improvement in HbA1c was observed among all the subjects, including the defaulters, from baseline until post-2, which was up to a year. Several studies reported similar findings. A study in three internal medicine clinics in Northeast Ohio showed a significant reduction in HbA1c in the pharmacist-led diabetes management group compared to the usual physician care group 6 months post-index [17]. This corresponded with a randomized clinical trial in the United States showing a significant reduction of 2.5% in HbA1c over 12 months of pharmacists’ care [18]. While the effectiveness of pharmacist-led diabetes mellitus management was unequivocally demonstrated, we further investigated different system and patient-related factors which predicted its effectiveness denominated by HbA1c level, which was not widely reported in the literature.

Notably, there was a significant difference in HbA1c reduction between DMTAC defaulters and non-defaulters. Patients who adhered to DMTAC follow-up were more likely to experience a greater magnitude of HbA1c reduction. Surprisingly, there was limited literature regarding the impact of adherence to diabetes clinic follow-up schedules on HbA1c levels. Two studies in the 1980s and 1990s reported that patients who defaulted to diabetes clinic follow-up had significantly poorer control of HbA1c and developed more vascular-related complications [19, 20]. A recent study consolidated those findings, further explaining the reasons for defaulting follow-up in diabetes clinics [21]. The most common reason for defaulting was multiple clinics and forgetting appointments due to other comorbidities [21]. In this study, eight out of ten patients had hypertension and dyslipidemia, while two out of five had at least one macro or microvascular diseases. This could indicate the need to treat diabetic patients with a more holistic and multidisciplinary approach by combining different clinics and reducing the number of follow-ups. The applicability of other approaches such as phone-call education, flexi-hours clinics, web-based virtual counselling, and digital self-monitoring applications should be examined to minimize default among diabetic patients.

While there was no difference in glycemic control between patients who visited DMTAC in hospitals and primary health clinics, we observed a higher default rate in hospital patients. This was similar to a previous local study, despite we observed a lower degree of differences [22]. In Malaysia, public hospitals are located in cities, while public health clinics are scattered to provide broad coverage of healthcare services, including rural areas [23]. Hence, primary health clinics are generally more accessible compared to hospitals. Furthermore, hospitals have a higher patient load and may require a longer waiting time than primary health clinics [24]. Considering the higher default rate in hospitals, there is a potential need to discharge patients with controlled HbA1c from hospitals to primary health care based on a set of pre-determined criteria [25]. It is therefore critical to strengthen primary health care through upgrading basic infrastructures and human resources, improve training and capacity of primary care staff, integrating hospital and primary care and enhancing community-based care to optimize its utilization among diabetes patients [26].

We further analyzed the predictors that led to HbA1c reduction. During the first phase of DMTAC follow-up (four to six months post-recruitment), more frequent DMTAC visits had a positive impact on glycemic control, whereas a longer duration of diabetes produced negative impact. Patients with a longer duration of diabetes exhibited poorer HbA1c control, which may be attributed to factors such as disease progression, cumulative metabolic effects, and the potential development of complications over time [27]. An increase in the frequencies of DMTAC visits enabled pharmacists to monitor patients’ responses more closely and hence optimize their antidiabetic regimen timely. Nevertheless, our findings contradict previous reports. Two local studies and a Japanese study revealed that patients who underwent intensive and less-intensive follow-ups demonstrated a similar level of glycemic control [12, 28, 29]. Interestingly, we did not observe such an impact in the second phase (eight to twelve months post-recruitment) of DMTAC follow-up. Taken together, more intensive follow-ups at the initial six months may benefit the patients and could be tapered down once the patients are stabilized to optimize human resources and treatment costs.

On the other hand, optimization of diabetic treatment significantly improved glycemic control in the longer time horizon of DMTAC follow-up. We found that pharmacists-led interventions, particularly dosage adjustment and the addition of antidiabetic agents, contributed significantly to HbA1c reduction. Our findings were substantiated by a randomized controlled trial in the primary health settings, which involved pharmacist-led dosage adjustment, drug-related problem detections, and alteration of medication regimen [30]. Javaid et al. reported that patients who received those interventions demonstrated better glycemic, blood pressure, and lipid control [26]. Diabetes treatment involves complex algorithms and regimens, necessitating individualized treatment plans, especially when involved insulin [31]. This was tested in another randomized control trial, where subjects on insulin treatment achieved superior glycemic control when they received intervention comprised of digital system integration in a blood glucose measuring device, which assists health providers in timely insulin dose adjustment [32]. The efficacy and feasibility of such a technologies-driven model warrant further investigation among diabetic patients in local settings.

To our best knowledge, this was the first real-world study that evaluated system and patient-related factors, including frequencies of follow-up, default rate, types of facilities, and interventions that was associated with improvement in glycemic control. This study has a few limitations. Due to the retrospective design, causality establishment was not warranted. Although patients were followed up for up to one year in our study, the longer-term efficacy of the DMTAC service remains uncertain. Therefore, future studies should investigate the impact of such programs on patients’ self-care behaviors and assess glycemic improvement over extended periods to evaluate the sustainability of the observed benefits. Further research investigating the factors and consequences of defaulting on diabetic clinics is warranted, specifically exploring tangible health outcomes beyond surrogate markers such as glycemic control.

## Conclusion

DMTAC services led to significant improvements in glycemic control across all subgroups. Default rate was higher in hospitals, and defaulters demonstrated poorer HbA1c control. More frequent DMTAC visits and specific pharmacist interventions resulted in improved glycemic control. However, the different roles of these predictors across the two DMTAC follow-up phases suggest the benefits of intensive early follow-up and tailoring medication regimens according to individual needs in long-term diabetes management. The findings underscore the vital role of pharmacists in diabetes care and offer guidance to policymakers for determining key performance indicators in DMTAC.

## Data Availability

The datasets generated and/or analyzed during the current study are not publicly available due to confidentiality of patients, but are available from the corresponding author on reasonable request.
